# The Book World of Medicine and Science

**Published:** 1900-03-03

**Authors:** 


### The Book World of Medicine and Science.

The Lute and Lays. By Charles Stuart Welles, M.D. (London: George Bell and Sons; New York: Macmillan and Co., Limited. Price 3s. 6d. net.)

Dr. Welles's poems are of very varied merit. "The Lute," which is by far the longest piece in the volume, is cast in the measure of "In Memoriam," and to write in that stanza is a rash thing for any poet to do after Tennyson. But here and there are lyrics which possess that indefinable touch which differentiates poetry from mere verse. Such is the little eight-line entitled "Roses," and in a deeper and tenderer strain, the longer one called "A Star of Evening." "A Song of Spring" has the charm of sincerity, but the companion "Song of Summer" is stiff and affected. Indeed, we think that what Dr. Welles needs is not the spirit of poetry but that of self-criticism. The author is an American, and the introductory poem to New England, written in London, is one of the most attractive pieces in the collection.

Spencer's Disease (Dermatitis Multiformis Exfoliativa). By Walter Spencer, M.D. (Privately printed. Pp. 15, 8vo.)

This pamphlet gives a descriptive account of an epidemic disease which, in 1898, prevailed in the wards of the Home of the Sydney Rescue Work Society. The disease appears to have been named Spencer's disease—we presume after the writer of this pamphlet, who is also physician to the Sydney Rescue Society, and observed the outbreak. The general characters of this exfoliative dermatitis seem not unlike, as Dr. Spencer says, those of the mysterious complaint which Dr. Savill described some years ago; but it certainly does not differ greatly from the disease described in 1870 by Ritter as epidemic amongst infants at Prague. Dr. Spencer's account is painstaking, and we think he establishes the infective nature of the complaint. But his mind seems scarcely of an ætiological bent, and in place of any bacteriological investigation we are supplied—as is the way of dermatologists—with various attempts in nomenclature. If Dr. Spencer has not made obscure things clear, he has at least contributed an important clinical record to dermatological literature.

Effects of Borax and Boracic Acid on the Human System. By Dr. Oscar Liebreich. A translation from the German. (London: J. and A. Churchill. 1899. Price 2s. Pp. 43, 4to.)

This brochure, from the pen of Dr. Liebreich, is an evident apology for the use of borax and boracic acid as food preservatives. It does not touch any of the wider questions involved, and makes no reference to what may be termed the "vital" effects of these substances. Nor does it attempt to defend the use of salicylic acid. But the author has made an enormous number of experiments on the bodies of lower animals, and, carefully collating the results, believes that he has demonstrated that borax and boric acid are really wholesome substances, and not injurious to the human system. We are not greatly concerned as to the accuracy of these experiments in themselves, and are in fact quite prepared to accept them. But we fail to see that Dr. Liebreich's results in any way determine the real question at issue, namely, the effect on the human organism of the continued ingestion of preserved foods. Doubtless it is true, as Dr. Liebreich states, that poodles and mastiffs to whom boric acid in solution is given gain weight under certain conditions. But what of the effect on hand fed babies when their daily food is invariably tainted with chemicals? The question is not one which can ever be settled by the experiments of chemists and physiologists, and the pseudo-science of experimentalists such as Dr. Liebreich will only bring about confusion and obscurity. The opinion of cultivated practitioners is almost invariably one of opposition to these preservatives, and the success or failure of their opposition will depend, not on academical discussion, but on the value of certain economic factors which control the production and distribution of our food supply. But in the meantime it is a pity that it should appear that in any degree medical science countenances food preservatives so long as there is a possibility of securing for the masses an adequate supply of what Dr. Poore has so aptly termed "living foods."

Merck's Manual of the Materia Medica. (E. Merck, Darmstadt. 1899. Pp. 212. 12mo.)

This little book, published by the well-known firm of chemists at Darmstadt, is obviously designed to fulfil several purposes. It is only necessary for us to allude to one of the publisher's aims, that to supply a need felt by every practitioner of readily refreshing his memory. To this end the subject matter is arranged in two sections; one, a categorical list of pharmaceutical remedies; the second, a tabulated list of drugs appropriate to certain conditions. The first list contains many names of synthetic products of but ephemeral notoriety; the second is well arranged and complete. But we are surprised to notice that in the case of this booklet, as in the case of two others that are in our hands, calcium chloride is not mentioned as an hæmostatic for either internal or external use. The book is attractively got up and is of little bulk, being printed on a kind of India paper.

Aids to Materia Medica. Part III. By W. Murrell, M.D., F.R.C.P. (London: Baillière, Tindall, and Cox. 1900. Pp. 92. 12mo. Price 2s. 6d)

This, the third and concluding part of Dr. Murrell's useful little book, deals with synthetical products, drugs of animal origin, glandular therapeutics, and serum therapeutics. For the sake of those yet unacquainted with the preceding parts, we may say that in these little books Dr. Murrell deals concisely and lucidly with such points concerning "Materia Medica" as may be of profit to the student. The subject-matter is arranged in sections, the sections treating separately of such topics as Wines and Spirits, the Salicylates, Tuberculin, and so forth. Incidentally a great deal of useful information is conveyed, and students will, we hope, appreciate the valuable and classical prescriptions occasionally introduced. We regret, however, that we are compelled to say that in our opinion Dr. Murrell is less happy in dealing with bacteriological and similar topics than in discussing the older materia medica. In fact, some of the bacteriological statements are deplorably crude. The account of immunity, for instance, is quite inadequate. The work is concluded with an arrangement of drugs in groups, which it is hoped may prove "useful to students preparing for examinations." We should be unable from these groups to learn that strychnine is a cardiac stimulant, or that potassium is a cardiac depressant. The terms "pulmonary stimulant" and "pulmonary sedative" are, to say the least, inept; and that Dr. Murrell should care to speak of copaiba and eucalyptus as "urethral alteratives" we are loth to believe. We are, too, always a little surprised that writers on therapeutics should continue to state that it is doubtful whether ergot, savin, and pennyroyal are abortifacients. There are few general practitioners who are not made aware many times in a year of the successful though illegitimate use of these drugs, though it may well be that the consultant and hospital physician meet with but few instances. In truth, this arrangement of drugs in groups is an unworthy one, and we sincerely hope will be little called for at any qualifying examination.

